# Gilles de la Tourette Syndrome—A Disorder of Action-Perception Integration

**DOI:** 10.3389/fneur.2020.597898

**Published:** 2020-11-26

**Authors:** Alexander Kleimaker, Maximilian Kleimaker, Tobias Bäumer, Christian Beste, Alexander Münchau

**Affiliations:** ^1^Center of Brain, Behavior and Metabolism, Institute of Systems Motor Science, University of Lübeck, Lübeck, Germany; ^2^Department of Neurology, University Hospital Schleswig-Holstein, Lübeck, Germany; ^3^Cognitive Neurophysiology, Department of Child and Adolescent Psychiatry, Faculty of Medicine, Technische Universität Dresden, Dresden, Germany

**Keywords:** Gilles de la Tourette syndrome, Theory of Event Coding, action perception binding, premonitory urge, ERP, RIDE

## Abstract

Gilles de la Tourette syndrome is a multifaceted and complex neuropsychiatric disorder. Given that tics as motor phenomena are the defining and cardinal feature of Tourette syndrome, it has long been conceptualized as a motor/movement disorder. However, considering premonitory urges preceding tics, hypersensitivity to external stimuli and abnormalities in sensorimotor integration perceptual processes also seem to be relevant in the pathophysiology of Tourette syndrome. In addition, tic expression depends on attention and tics can, at least partly and transiently, be controlled, so that cognitive processes need to be considered as well. Against this background, explanatory concepts should encompass not only the motor phenomenon tic but also perceptual and cognitive processes. Representing a comprehensive theory of the processing of perceptions and actions paying particular attention to their interdependency and the role of cognitive control, the Theory of Event Coding seems to be a suitable conceptual framework for the understanding of Tourette syndrome. In fact, recent data suggests that addressing the relation between actions (i.e., tics) and perceptions (i.e., sensory phenomena like premonitory urges) in the context of event coding allows to gaining relevant insights into perception-action coding in Tourette syndrome indicating that perception action binding is abnormally strong in this disorder.

## Clinical Phenomenology, Therapy and Previous Directions of Research

Gilles de la Tourette syndrome (GTS) is a childhood-onset multifaceted neuropsychiatric disorder defined by several motor and at least one phonic tic, lasting for no <1 year (1) First symptoms usually occur as motor tics around the age of 6, commonly affecting the face, head and neck (2). In most cases, first phonic tics occur several years later (2). Importantly, GTS is characterized by a repertoire of repetitive tics, which is relatively stable at a given time but fluctuates considerably over longer time periods (2, 3). Tics range from simple movement/sounds like eye blinking or rolling, sniffing, throat clearing or grunting to complex movements/vocalizations including squatting, head and body turning or twisting, utterances of words or, in rare cases, phrases, which can be obscene (4). Tics are sometimes difficult to distinguish from spontaneous movements in healthy controls (4, 5). However, tics occur in a repetitive pattern and appear temporally and situationally misplaced (6).

GTS patients often have psychiatric comorbidities, particularly attention deficit hyperactivity disorder (ADHD, about 60%) (7) and obsessive-compulsive disorder (OCD, about 40%) (8). Clinical course is benign in most cases with symptom maximum about the age of 10–11 followed by considerable improvement or freedom of symptoms in late adolescence or early adulthood (2, 9, 10). Thus, in most cases, besides counseling, no specific treatment is needed. However, symptoms persist into adulthood in about 20% of cases (11), can deteriorate significantly and may impair the quality of life of affected patients (12, 13).

In children or adults requiring more specific treatment in addition to counseling or general measures including stress reduction and measures at school or at work, different therapeutic approaches are available encompassing psychotherapy (14), particularly habit reversal therapy (15), pharmacotherapy (antipsychotic medication, alpha-2-agonists) (16, 17) and botulinum toxin injections (17, 18). However, in a few cases, these measures are insufficient or cause intolerable side effects. Then, deep brain stimulation, particularly of the internal segment of the globus pallidus, might become an option (19).

Research on GTS and its pathophysiology goes back into the late 19th century. GTS was initially described by the French neurologist and coroner George Gilles de la Tourette. It was first considered a disorder of predominantly psychiatric or psychosomatic origin (20). This view changed in the 70ies and 80ies of the last century, mainly because of advances of neurophysiological research (21, 22) and effectiveness of antipsychotic drugs (16). GTS has since been regarded an organic neurodevelopmental neuropsychiatric disorder and it has become clear that its etiology is largely genetic (23, 24). A wealth of data has accumulated indicating that structural and functional abnormalities in the basal ganglia (e.g., a volume reduction of the striatum) (25, 26), in cortico-striato-thalamo-cortical circuits (27, 28), and in some cortical areas including medial-frontal regions and the prefrontal cortex (26, 29) as well as hyperactivity of the dopamine system (30–32) are core findings in GTS. However, despite of these data documenting various abnormalities on a neuroanatomical, neurophysiological and brain imaging level (6), there is so far no unequivocal comprehensive theory or framework providing an explanation for GTS with its multiple facets.

Besides tics, sensory phenomena, particularly premonitory urges, represent a core feature of GTS (33). These sensations typically precede tics (33). Their characteristics are very diverse. In addition to the feeling of an urge to perform a certain action, of anxiety or restlessness (33) also more localized somatic sensations including an itch, tinge, ache or numbness can occur (34). The execution of tics typically leads to a temporary decrease of premonitory urges, whereas their suppression causes an increase (2). Furthermore, echophenomena, i.e., automatic imitation of observed words (echolalia) or actions (echopraxia) as well as coprophenomena, i.e., the execution of obscene gestures (copropraxia) or, as pointed out above, the utterance of swearwords (coprolalia) are characteristic features (35, 36). Although the latter dominate public perception, particularly in Social Media, they are only present in about 20% of cases (35). Importantly, cognitive processes seem to have great impact on tic severity. For instance, stress and focusing on tics lead to an increase of symptoms, whereas distraction ameliorates them (37, 38).

Against the background of clinical phenomenology and data on pathophysiology, an explanatory approach encompassing not only the motor sign tics, but also abnormalities within the somatosensory system as well as cognitive control processes would be needed. In this manuscript, we will outline such a framework taking into account novel behavioral and neurophysiological findings in GTS patients corroborating its validity.

## The Role of Perceptual Processing in GTS

Motor and phonic tics are the most salient feature of GTS. Thus, one might argue that GTS mainly represents a motor/movement disorder characterized by abnormalities in action control. However, there is increasing evidence for perceptual and cognitive processes to play a crucial role as well.

First and foremost, abnormalities on a perceptual level are evidenced by premonitory urges preceding tics. These are described as unpleasant and disruptive and might interfere with attention and concentration (39). Some GTS patients consider their tics as voluntary actions in reaction to their premonitory urges (34). Further, premonitory urges are increased by tic suppression (2). Thus, tics and premonitory urges seem to be mutually dependent. However, it should be noted that some GTS patients do not have urges at all or only occasionally. This is the case in about 10% of adult patients with GTS (40). Also, urges are reported in about 25% of 8–10-year-old children with GTS but nearly 60% of 15–19-year-old adolescents with GTS (41). In line with this, there are studies showing that the probability of the occurrence of premonitory urges increases with age (34, 39). Leckman et al. found that the average age children become aware of premonitory urges was 10 years (33). Therfore, it might be argued that urges represent a response to having tics rather than a tic-driving phenomenon. However, facing the close clinical interdependency of tics and premonitory urges (see above) it seems reasonable that these two phenomena are closely interlinked within the pathophysiology of GTS. Thus, the fact that especially younger GTS patients do not report premonitory urges does not necessarily mean that they are absent but rather that young GTS patients are not aware of them due to altered perceptional structures.

Premonitory urges though are not the only argument for altered perceptual processing in GTS. Besides premonitory urges, there is hypersensitivity to external stimuli in many GTS patients, i.e., patients are unusually aware of stimuli most people would not recognize, e.g., skin contact with cloths or contact with a chair, often leading to irritation and distraction (42). This is not due to altered sensory perception thresholds. Thus, quantitative sensory testing (43) comprising different sensory parameters including thermal, mechanical and pain thresholds was normal in GTS patients (44). Therefore, hypersensitivity to external stimuli seems to be due to alterations of central processing of perceptual information.

Altered processing of perceptions is also documented by abnormal pre-pulse inhibition (PPI). PPI referes to a startle reaction, typically caused by an acoustic stimulus, measured by means of ocular muscle contraction assessed using EMG, which is attenuated by a preceding, subliminal acoustic stimulus (“pre-pulse”) (45). PPI has been found to be reduced in GTS (46). In line with this, there are alterations of short afferent inhibition. Short afferent inhibition tested by delivering electric current to the median nerve at the fingers or at the wrist prior to a transcranial magnetic stimulation pulse applied to the hand area of the contralateral motor cortex (47). The amplitude of the motor evoked potential elicited by the transcranial magnetic stimulation pulses is reduced by median nerve electrical pulses provided the latter are given between 20 and 25 ms earlier (47). Since short afferent inhibition was shown to be diminished in GTS (48, 49), this again provides evidence for abnormal central processing of perceptual input. Grip force experiments showing that GTS patients used higher grip force to hold an object with defined weight compared to healthy controls (50) confirm this hypothesis.

Taken together, premonitory urges, hypersensitivity to external stimuli and abnormalities in sensorimotor integration (i.e., PPI, short afferent inhibition and grip force experiments) suggest that perceptional processing is altered in GTS and that the relation of these perceptual processes to motor phenomena and actions is of importance.

## Perception-Action Integration—A Cognitive Approach Based on the Theory of Event Coding

The Theory of Event Coding (TEC) introduced by Bernhard Hommel (51), represents a comprehensive cognitive framework for perception-action coding paying particular attention to the interdependency of perception and acting. TEC makes the strong assumption that perception and action are not isolated parts of human information processing but rather interconnected elements in a common representational format. This is why TEC is also known as “common coding theory.” TEC assumes that perceptions such as visual, somatosensory or auditory stimuli are stored in so called “object files,” whereas actions are stored in “action files” (51). Importantly, perceptions and actions are not stored and processed separately in discrete functional units, but are rather related to each other. This means that once perceptions (“object files”) and actions (“action files”) occur at about the same time, this leads to a coupling, or binding, between them (51). “Action file” and related “object file” are thus bound in an “event file” (51). According to the “common coding” nature of TEC, “action files” and “object files” share common neural codes (51). Whenever a previously established “event file” is activated by one of its features / elements, the entire “event file” is retrieved according to a “pattern completion logic” (52). Thus, once a feature (perception or action) is stored in an “event file,” it can no longer be processed separately (51). Of note, preceding bindings within an event file strongly affect subsequent actions, which can lead to behavioral costs (53–58). More precisely, as soon as different features of an action or object are bound, these features are processed concomitantly, so that processing a new combination of the same features requires time-consuming unbinding and rebinding processes (53). It follows that lower costs (i.e., superior performance) are to be expected if either the complete “event file” is reactivated and processed or a completely new “event file” is established. Higher costs (i.e., worse performance) are expected, if there is a partial overlap between features. Thus, partial repetition of features of an “event file” entails worse performance concerning the accuracy and reaction time in stimulus response tasks compared to conditions, where all features or no feature are iterated (“partial-repetition-costs”). These “partial-repetition costs” can serve as a measure of the strength of perception-action bindings (59).

Since the environment offers a wealth of sensory input with the majority being irrelevant to the intended action, there must be a cognitive mechanism of weighting it in terms of relevance for the intended action. Otherwise, if all perceptions and actions perceived at a given time were stored into event files equally without paying attention to their task relevance, a link between intended action and perceptual information derived from it would not be possible and therefore goal directed actions would not be feasible. The mechanism of increasing the weight of features that are coded on task-relevant dimensions is referred to as intentional weighting (60). It entails that during preparation of a task stimulus dimensions that have been experienced or are assumed to be important for the task are not only increased in weight but are also stored in event files more effectively (60). In other words, preparing for a task involves the priming/activation of task-relevant feature dimensions. On an experimental level, the importance of cognitive processes in the form of intentional weighting is shown by voluntary event file coding paradigms. Whereas, automatic event file coding is based on stimulus response tasks combining perception and action as a function of their close temporal relation, voluntary binding increases the relevance of certain stimulus features, for instance by explicitly asking probands to pay attention to them (53).

Summing up, representing a cognitive framework for perception and action, TEC appears as a suitable framework for investigating GTS in a comprehensive way considering clinical phenomenology, i.e., tics and somatosensory phenomena including premonitory urges, hypersensitivity to external stimuli and cognitive processes relevant for the understanding of GTS.

## The Neural Basis of the Theory of Event Coding

A number of studies have examined the neuronal mechanisms underlying feature binding according to TEC. It has been suggested that perceptual categorization and attentional selection processes play an important role for integrating different feature codes into an object file (59). Such feature integration processes occur along the dorsal visual stream (61), with the parietal cortex and neural oscillations in the alpha and gamma frequency band playing a crucial role (62, 63). In addition, frontal and fronto-parietal networks are also relevant for visual feature integration (62, 64, 65). Regarding event files, the role of the dopaminergic system has been investigated extensively using pharmacological, molecular genetics and substance abuse approaches (66–69), as well as manipulations of reward anticipation (70). These studies suggest that event file coding is strongly modulated by the dopaminergic system.

Several studies have also addressed the functional neuroanatomical network involved in event file coding. Using functional brain imaging and non-invasive brain stimulation, it has been shown that event file coding is mediated via a widely distributed network including the supplementary motor area, the dorsolateral prefrontal cortex, hippocampus and parahippocampal gyrus (71–73), suggesting that perceptual and/or attentional mechanisms as well as response selection and memory encoding are relevant. The parahippocampal gyrus has also been suggested to be involved in response inhibition processes (74), especially when response inhibition processes are not successful and corrective actions via error monitoring are needed (74, 75). This suggests that cognitive control processes play a role in the automatic retrieval of event files. Underlining this, Kühn et al. suggested that automatic retrieval entails a conflict with ongoing response selection (72). Medial prefrontal structures are likely to be relevant for event file coding as well, as these have frequently been shown to be involved in conflict monitoring (76–78).

A number of electrophysiological studies examined the neurophysiological mechanisms related to event coding. For instance, Keizer et al. (79) studied the interrelation between the brain's electrical activity recorded from the cortex and event file coding. This electric activity typically exhibits oscillations at different frequency spectra, for instance theta band activity (5–7 Hz), beta band activity (12–20 Hz) or gamma band activity (36–44 Hz). Keizer et al. showed that a neurofeedback training designed to increase gamma band activity or beta band activity had an impact on event file binding. Enhancing gamma band activity led to greater flexibility in retrieving episodic bindings, which points to a role of gamma band activity in top-down control. In another study (79) it was shown that also feature binding (in the sense of object files) is modulated, with higher gamma band activity being related to decreased binding costs. The modulation of temporally dissociable cognitive-neurophysiological sub-processes from perception to response selection during event file processing was examined using event-related potentials (ERPs), also addressing the importance of the dopaminergic system by introducing a reward manipulation (70). It was shown that neurophysiological correlates of processes of stimulus categorization (reflected by the P1 component of the ERP) (80, 81) are modulated, but not attentional selection processes (reflected by the N1 ERP) (82). N2 ERPs were modulated across difficulty levels of stimulus-response association unbinding. The source localization analysis showed that the N2 amplitude modulations were related to the anterior cingulate cortex, suggesting that feature unbinding is primarily a function of this area (83, 84). Moreover, the P3 ERP was modulated, which was related to activation differences in the inferior parietal lobe (BA40), i.e., the temporo-parietal junction. This region has previously been shown to be related to modulations in the P3 component (85) and is also considered crucial for response selection (86–89). Thus, processes underlying N2 and P3 modulations reflect distinct aspects of event file coding. One process seems to reflect feature unbinding (N2), while the subsequent process (P3) reflects mechanisms to select the appropriate response.

In a recent study, the network architecture of event file coding was examined (56). The key finding was that binding processes were reflected by small-world network characteristics in the theta band. When a previously established stimulus response binding facilitated a response, a higher grade of organization was seen in the network. This suggests that binding processes are reflected by networks connecting different assemblies of neurons in the theta frequency band with an increase in organization during binding processes.

## Tourette Syndrome in the Context of the Theory of Event Coding

Given that premonitory urges typically represent a mounting unpleasant sensation enforcing tic execution and since tic execution typically attenuates premonitory urges (see above), there seems to be a strong interrelation between tics and premonitory urges (90). Altered internal monitoring (91) as well as an increased sense of agency (92) in GTS patients further underpin the need for a concept encompassing perception and action. TEC supposes that performing a predefined action both is based on and produces perceptions (59). In the context of TEC, the motor parts of tics might thus be considered as actions (stored in “action files”), premonitory urges as perceptions (stored in “object files”) (90); tics as a whole would then represent perception-action phenomena, i.e., “event files.” Given an apparently tight link between object files (urges) and action files (tic related motor output), bindings within event files, i.e., between object and action files, are expected to be primarily abnormal, i.e., increased, in GTS, whereas binding in object files should not be increased (40, 90).

In the first study testing the TEC concept directly in GTS patients (93), an established object file behavioral paradigm was used (53). Participants were faced with three white rectangular boxes, vertically arranged on a black background. Vertical and horizontal lines in red or green were presented in the top or bottom box as stimulus (S) 1 (S1) and S2. The stimuli thus had three feature dimensions (orientation, color and position). In each trial, subjects were required to carry out two responses (R) (R2 and R3). They were first asked to respond to S2. After R2, R3 had to be carried out, where subjects were tested for their memory of one of the features of S1. Participants were asked about (a) the orientation, (b) the color, or (c) the location of S1. There were two response alternatives for each question and participants could indicate their response by pressing one of two response buttons. The features (i.e., orientation, color and location) could vary between S1 and S2, creating conditions with full feature overlap (i.e., 3 features), partial feature overlap (i.e., 2 or 1 overlapping features) and no overlap between features of the S1 and S2 stimuli. In healthy controls, performance deteriorated with increasing overlap between stimulus features in a given trial, which was explained with a feature binding/unbinding concept in the framework of TEC (59, 94). In contrast to healthy controls, performance was unaffected by varying feature-overlap levels in GTS (93), indicating that binding of perceptual features into an object file is weaker in these patients. There were no correlations with clinical characteristics, in particular with tic severity. Therefore, altered perceptual processing in GTS probably reflects a facet of this disorder that is not directly related to tics. Of note, deficits in visuo-motor integration have been described in children and adolescents with GTS (95, 96). The results suggested that mechanisms underlying the processing of different aspects inherent to visual stimuli differ between GTS patients and healthy controls. Fronto-parietal connections are particularly important for visual feature integration (62, 64, 65). Weaker binding between visual features in GTS might be related to reduced structural and functional long-range connectivity in frontoparietal networks in these patients (97, 98). The results could also be interpreted in the context of abnormal awareness of volitional action in GTS (91, 99, 100). As outlined above, activation of event files and object files follows a pattern-completion logic (52). Activating one element of a network will automatically lead to activation of other network elements the extent and strength of which depend on the task relevance of priming, i.e., to what extent priming is intentionally (voluntarily) weighted (52). In the task used in the study by Beste et al. (93), object file coding was voluntarily weighted because all features of the first stimulus had to be remembered. Given that awareness of volitional action has been shown to be reduced in GTS (99, 100) it is possible that this is the reason why “voluntary” binding was also weaker in them.

The hypothesis that bindings within event files, i.e., between object and action files might be stronger in GTS (40, 90), was tested in three studies using different methodology.

In the first study, GTS patients and healthy controls were asked to perform facial movements triggered by acoustic stimuli (101). These movements could either be tic-like movements being part of the tic repertoire of the patients or non tic-like movements. Parallel to movement execution, videoclips were displayed on a screen either presenting the same movement being executed, i.e., compatible movements, or other movements, i.e., incompatible movements. In healthy controls and in GTS patients when executing non-tic like movements, reaction times were increased when incompatible videoclips were displayed. Importantly, when GTS patients performed tic-like movements, incompatible videoclips did not entail increased reaction times, i.e., did not interfere with tic-like movements (101). This could be interpreted such that binding between tic-like movements and acoustic stimuli in GTS patients are stronger compared to non tic-like movements in patients and bindings in healthy controls leading to GTS patients being less prone to distraction in case tic-like movements are executed.

In the second study, Petruo et al. (54) carried out a unimodal vs. bimodal visual/acoustic Go/NoGo paradigm in adolescents with GTS. The Go-task comprised the German word “Drück” (“press”) presented on a computer screen demanding the execution of a certain keypress. Within the NoGo-task the word “Stop” was displayed, i.e., the keypress had to be withheld. Further, in some of the trials, visual stimuli were accompanied by acoustic stimuli (“Drück” or “Stop”) presented simultaneously that could be compatible or incompatible. Accordingly, the experiment exhibited 6 different conditions: Go-task without acoustic stimulus, Go-task with compatible acoustic stimulus, Go- task with incompatible acoustic stimulus, NoGo-task without acoustic stimulus, NoGo-task with compatible acoustic stimulus and NoGo-task with incompatible stimulus. The performance differences between unimodal and bimodal stimuli were significantly larger in GTS patients compared to healthy controls. More precisely, the rate of false alarms (keypress at stop-signal in NoGo-tasks) was higher when inhibitory control had to be exerted in case of unimodal visual stimuli. Hence, there seems to be increased binding between bimodal stimuli and responses leading to increased costs when switching between responses instructed by bimodal and those instructed by unimodal stimuli. The neurophysiological (EEG) data demonstrated that this was related to perception-action binding processes in the right BA40 (54).

**Figure 1:**
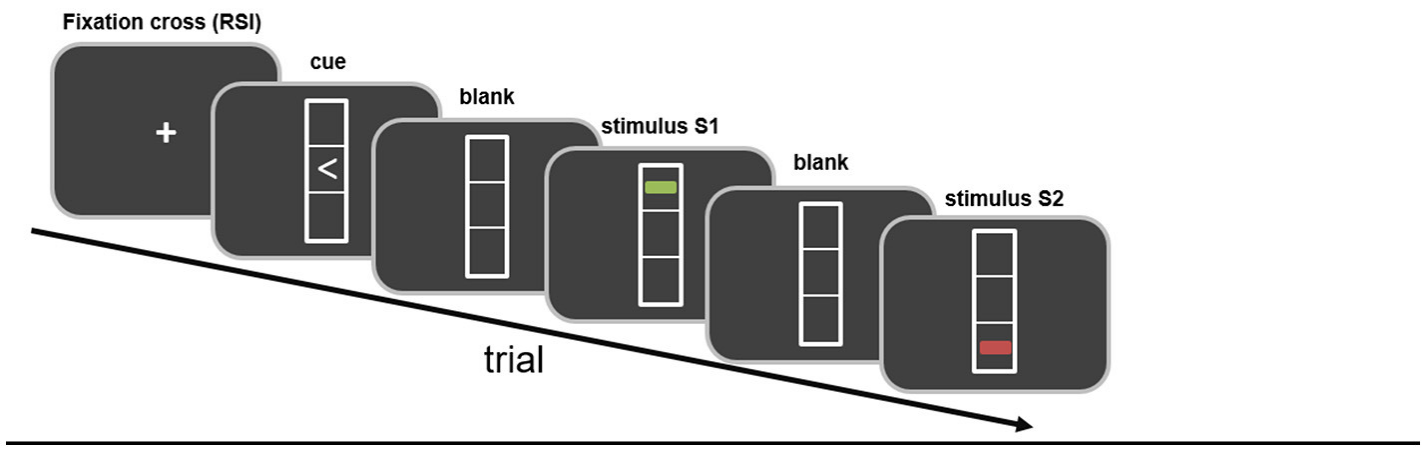
Schematic illustration of the paradigm. Stimuli and their temporal sequence are shown. Further details are given in the main body of the text.

In the third study, Kleimaker et al. directly addressed perception-action binding in GTS in the context of TEC (102). The strength of perception-action binding was measured using a previously established visuo-motor event file task instructing left or right key presses by means of visual instructions displayed on a computer screen (see above) (53). This paradigm has been developed from the object file outlined above. It begins with a cue signal in the form of a left- or right-pointing arrowhead instructing a right or left key press (reaction 1, R1). Importantly, the cue signal is not supposed to be answered immediately. Instead the response should be withheld until a second stimulus (stimulus 1, S1) appears. S1 is a multi-feature dimension stimulus as described above presented by a line displayed in three different dimensions (color: red/green, orientation: vertical/horizontal, position: top/down). Even though the execution of R1 is triggered by the appearance of S1, it is carried out regardless of any of the features of S1 but instructed by the direction of the arrowhead of the cue stimulus (see Figure 1). In this way, action (R1) and perception (S1) occur at about the same time resulting in a binding between them, i.e., an “event file” is established. This is followed by a third stimulus (stimulus 2, S2) with the same feature dimensions as S1. S2 needs to be responded to directly by performing a key press depending on one of the features of S2 (color, orientation or position). In case stimulus features and responses match between S1/R1 and S2/R2 or do not overlap at all, processing and responding to S2 is compatible with the created “event file,” i.e., that no partial repetition costs are to be expected. However, in case the same stimulus features are associated with a different response or vice versa, the previously established “event file” needs to be resolved and a new one needs to be established resulting in partial repetition costs, i.e., higher error rates or slower reaction times. These partial repetition costs serve as a measure for the strength of perception action binding.

**Figure 2:**
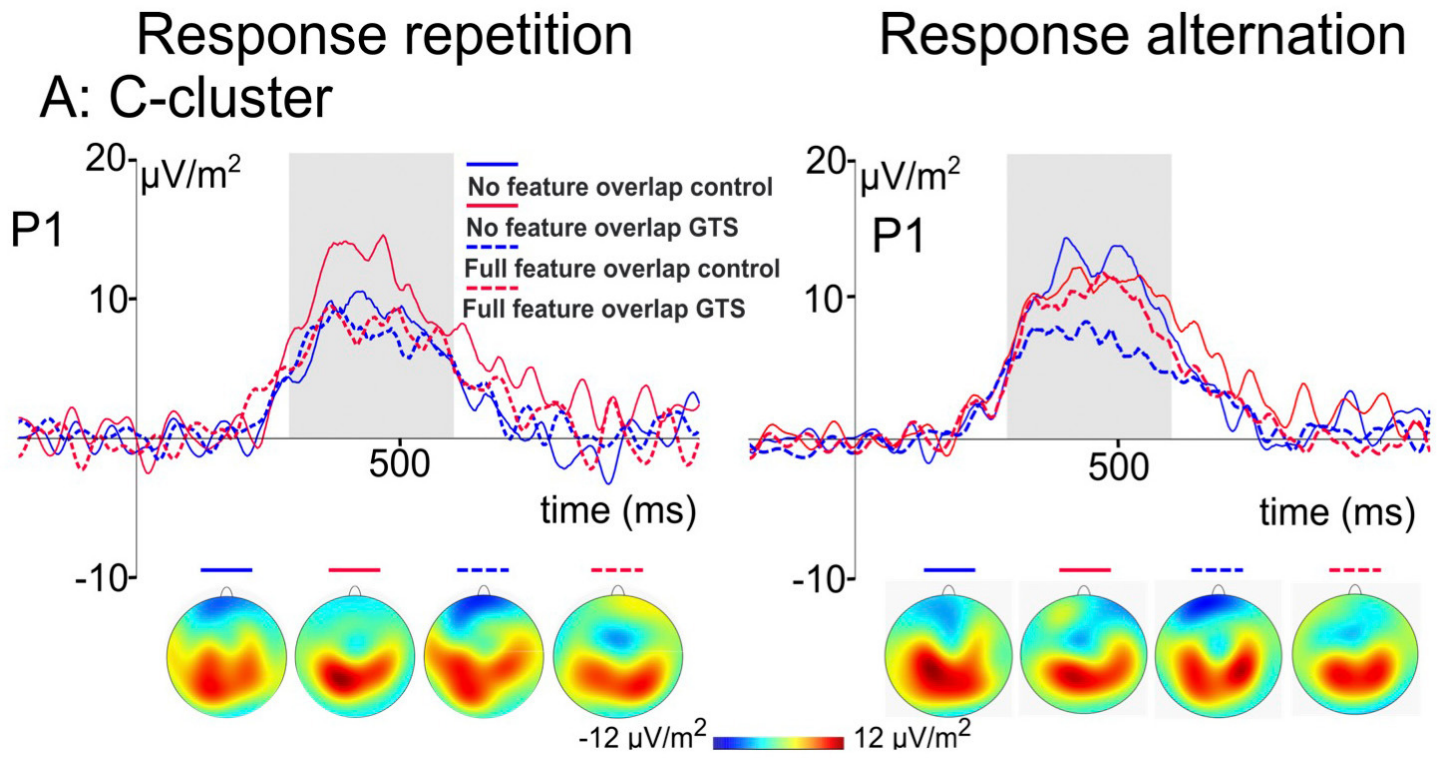
C-cluster ERP signal of the two-step stimulus response task by Kleimaker et al. (see above) derived by means of RIDE analysis. Using response repetition condition (left side) and response alternation condition (right side) as well as stimulus features being iterated (dashed line) or altered (solid line), there are conditions exhibiting partial repetition costs (e.g., response repetition in case of stimulus features being altered) and partial repetition benefits (e.g., response alteration in case of stimulus features being altered). Corresponding c-cluster ERP signals are compared between GTS patients (red lines) and healthy controls (blue lines). The scalp topography plots show the distribution of the mean activity in the analyzed time window.

Kleimaker et al. studied 24 GTS patients and 24 gender- and age-matched control subjects using this paradigm. As hypothesized, significantly higher partial repetition costs were found in GTS patients. Thus, this study for the first time yielded direct and strong evidence for increased perception-action bindings in GTS. In addition, the strength of perception-action bindings positively correlated with tic frequency at that time. Importantly, this suggests that increased binding strength is not an epiphenomenon in GTS but rather represents a core feature (102). Behavioral data were corroborated by event related potential findings during EEG. As regards event file coding the N2 component representing processes linked to conflict monitoring and feature unbinding (70) and the P3 component linked to response selection and rebinding processes (70) are of particular interest. N2 amplitudes have been shown to increase with increasing degree of feature overlap, i.e., more complex trials, whereas P3 amplitudes decreased when feature overlap increases, i.e., when unbinding processes are more complex (70). Of note, like other ERP components, N2 and P3 segments consist of different sub-components. More precisely, these components encompass signals predominantly derived from stimulus processing referred to as S-cluster, signals primarily engaged in response preparation and execution (R-cluster) and processes of stimulus response interaction (C-cluster) (103). Due to intraindividual differences in latencies, these sub-components cannot readily be separated (104). A means to discern them is referred to as residue iteration decomposition analysis (RIDE) (104). Using timing and variability of these components, RIDE allows to separating standard ERP signals into their subcomponents. Thus, RIDE serves as a measure of controlling for intraindividual variability and isolates different coding levels in a theoretically meaningful way (103, 105). C-clusters best represent stimulus response association processes (106–108). They are modulated by processes linked to inferior parietal regions, particularly BA 40 (108) shown to exhibit functional altered in GTS (109, 110). In the study by Kleimaker et al. (102), standard ERP components, in particular the N2 and P3, did not differ between GTS patients and healthy controls. However, the RIDE analysis showed that whereas there were no group differences in the S- and R-clusters of the N2- and P3 components, C-cluster P3 differed (Figure 2). In healthy controls, amplitudes decreased in more difficult trials where rebinding processes were more complex. This was not the case in GTS, which can be interpreted such that cognitive control resources to resolve previously bound event files are not adequately recruited, so that problems arise when such perception action bindings need to be re-configurated (102).

Using standard low resolution brain electromagnetic tomography (sLORETA) algorithm (111), these effects were located in the left inferior parietal cortex (BA40). Thus, for the first time, it was possible to associate clinical, behavioral and neurophysiological findings of increased perception-action binding with a certain brain region in GTS. This might offer new avenues for experimental treatments. For instance, it appears plausible to target BA40 for non-invasive brain stimulation, e.g., repetitive transcranial magnetic stimulation.

As to the neural basis of increased binding shown in the study of Kleimaker et al. (102), increased connectivity in short-range sensorimotor pathways in GTS might play a role (98). More specifically, there is enhanced structural connectivity between striatum/thalamus and primary motor and sensory cortices, supplementary motor area and parietal cortices, which positively correlated with tic severity (28). Corroborating these findings, greater structural connectivity within the right motor cortico-striatal network in GTS patients was related to stronger engagement in habitual responses in a learning paradigm, both of which were correlated with greater tic severity (112).

Taken together, what emerges in GTS is a dichotomy of weaker binding in object files (93), probably explained on the basis of *reduced* long-range connectivity including fronto-parietal networks, but unrelated to tics *per se*, and increased event file binding (102), presumably caused by *increased* connectivity in basal-ganglia cortical projections, correlating with tic severity and a tendency for habit formation.

Findings related to the usefulness of TEC as an explanatory framework for GTS are also relevant for the understanding of the mechanisms of behavioral interventions. For instance, in a recent study, the effects of comprehensive behavioral intervention for tics on perception-action binding during inhibitory control were investigated (113). It was shown that the intervention altered inhibitory control in a condition where reconfigurations of perception-action bindings were necessary to perform inhibitory control. Comprehensive behavioral intervention for tics reduced increased binding between perception and action in GTS and thereby increased the ability to perform response inhibition. The results are the first to provide insights as to why comprehensive behavioral intervention for tics is effective by relating elements of this intervention to an overarching cognitive theoretical framework on perception-action bindings.

## Theory of Event Coding and Tourette—Misgivings and Limitations

TEC can explain characteristic GTS features including the relation of tics and urges or the suppressibility and suggestibility of tics. In which way the temporal course of the disease, including a relatively stable tic repertoire at a given time and fluctuations over longer time periods (see above) might be related to TEC is currently unclear. The fact that premonitory urges increase with increasing age (see above) might be related to developmental trajectories of event file coding but details need to be delineated.

Further, a possible key criticism relates to the question of whether TEC has any added value, i.e., whether is extends or complements already existing concepts regarding the pathophysiology of GTS. Thus, it might be argued that given that abnormalities in the basal ganglia and cortico-striato-thalamo-cortical are undoubtedly key findings in GTS further, i.e., alterative explanatory approaches are not required but are rather superfluous.

We would like to point out that key assumptions related to tics in the context of TEC can be reconciled with the established role of the basal ganglia, cortico-striato-thalamo-cortical circuits, and the dopaminergic system in the pathophysiology of GTS (90). Thus, the basal ganglia and cortico-striato-thalamo-cortical loops are very relevant for action selection depending on the relative salience of competing actions and also the integration of different sensory processes for action selection (114). Of note, the striatum contains a large number of neurons sensitive to sensory inputs (115). Therefore, striatal processes are likely playing an important role in perception action binding (90). This is to say that in principle ideas on the role of the basal ganglia and cortico-striato-thalamo-cortical in GTS and the concept of TEC as a framework for GTS are not mutually exclusive. More specifically, an important concept of how tics may be generated relates to the model of motor pattern generators (MPG) assuming that the basal ganglia serve as a “brake” on MPGs and that loosening of this brake may lead to the execution of a movement/action (116). It has been argued that in GTS braking mechanisms within the basal ganglia are defective with the consequence that aberrant activations of the MPGs are not withheld but manifest as tics. Such an MPG model and TEC can be reconciled.

The MPG model and TEC differ in that the former focuses on motor phenomena and processes and does not account for antecedent processes leading to aberrant activation becoming apparent as tics (90). The added value of TEC might be that it extends such a motor-centered perspective to include antecedent mechanisms leading to the formation of activity foci; in other words, how these are established. The MPG model allows to explain activity changes of the MPG, for instance through altered dopaminergic transmission, but makes no assumptions as to the occurrence/formation of activity foci, which requires input, including sensory signals, into the striatum. Such input and its effect on or integration with subsequent actions/motor responses are conceptualized in the TEC framework and this is why TEC might complement and extend the MPG model (90).

Another concern relates to the specificity of perception-action integration abnormalities in GTS in the context of TEC. Because currently, TEC has not explicitly been tested experimentally in other neuropsychiatric conditions, or movement disorders, it cannot be stated with certainty that TEC related findings in GTS are in fact disease-specific. For instance, it is conceivable that obsessions and compulsions in OCD might share not only phenomenological features but also underlying neural processes with premonitory sensations and tics in GTS. Both OCD and GTS could be conceptualized as disorders of increased perception/action binding and disturbed unbinding, i.e., response switching. In OCD, stereotyped checking compulsions or cognitive rituals like compulsive counting may be viewed as event files. They are triggered by perceptions of incompleteness and “not-just-right” feelings associated with tension and anxiety. Patients with OCD are impaired in calibrating and adapting the relation between expected and actual outcomes of actions (117). Indeed, deficits in action monitoring have been documented in these patients (118). This may lead to uncertainty, excessive action monitoring and perseverative tendencies favoring habitual rather than empirical choices in cognitive paradigms with changing contingencies. Given their problems to adapt actions, OCD patients might represent a model for reduced response switching capacities. Viewed in the TEC context, perseverations in OCD could be the consequence of increased perception-action binding.

As to ADHD, sensory over-responsivity has been reported (119), which translated into TEC might point toward alterations in the formation, or composition, of objects files. This might also be true for patients with autism spectrum disorders, in whom increased sensitivity to external stimuli is a very characteristic feature (120).

## Summary

Given sensory phenomena including premonitory urges and hypersensitivity to external stimuli, altered sensorimotor integration and symptom dependency on stress and attention in GTS it is plausible to conclude that GTS is not a pure motor disorder. Representing a cognitive framework for perception-action processes, TEC seems very useful as an explanatory concept for the understanding of GTS. In keeping with clinical reasoning of an increased binding between actions (e.g., tics) and perceptions (e.g., premonitory urges) recent data from experimental studies including those conducted within a TEC framework suggest that GTS might be conceptualized as a disorder of perception-action integration.

## Author Contributions

AK and MK: writing of the first draft. TB, CB, and AM: review and critique. All authors contributed to the article and approved the submitted version.

## Conflict of Interest

The authors declare that the research was conducted in the absence of any commercial or financial relationships that could be construed as a potential conflict of interest.

## Abbreviations

ADHD: attention deficit hyperactivity disorder
ERP: event-related potentials
GTS: Gilles de la Tourette syndrome
MGP: motor pattern generator
OCD: obsessive-compulsive disorder
PPI: pre-pulse inhibition
R: reaction
RIDE: residue iteration decomposition analysis
S: stimulus
sLORETA: standard low resolution brain electromagnetic tomography
TEC: theory of event coding.
